# The effect of intraocular pressure elevation and related ocular biometry changes on corneal OCT speckle distribution in porcine eyes

**DOI:** 10.1371/journal.pone.0249213

**Published:** 2021-03-26

**Authors:** Marcela Niemczyk, Monika E. Danielewska, Malgorzata A. Kostyszak, Daniel Lewandowski, D. Robert Iskander

**Affiliations:** 1 Department of Biomedical Engineering, Wroclaw University of Science and Technology, Wroclaw, Poland; 2 Department of Mechanics, Materials and Biomedical Engineering, Wroclaw University of Science and Technology, Wroclaw, Poland; Bascom Palmer Eye Institute, UNITED STATES

## Abstract

The aim of this study was to evaluate the influence of increase in intraocular pressure (IOP) and cooccurring changes in ocular biometry parameters on the corneal optical coherence tomography (OCT) speckle distribution in *ex-vivo* experiments on porcine intact eyes. Twenty-three eyeballs were used in the inflation test where IOP in the anterior chamber was precisely set from 10 mmHg to 40 mmHg in steps of 5 mmHg and where eye biometry was utilized (IOL Master 700). To assess the influence of the duration of the experiment on the OCT speckle statistics, the second experiment was performed with 10 eyeballs at the constant IOP of 15 mmHg. Based on the OCT scans of central cornea (Copernicus REVO), spatial maps of the scale parameter (*a*) and the shape parameter (*v*) of the gamma distribution speckle model were estimated. The means of both parameters for each spatial map were computed within the 2 mm of the central stroma. Both distributional parameters statistically significantly varied with IOP and time (one way repeated measures ANOVA, all *p*-values < 0.001). The *a* parameter revealed a faster statistically significant increase in IOP up to 25 mmHg, regardless of time. Central corneal thickness (CCT), the anterior chamber depth, and the mean equivalent spherical power varied significantly with IOP, whereas CCT and axial length changed statistically significantly with time. Statistically significant correlation was found between CCT and the *a* parameter, after removing IOP as a confounding factor (r = −0.576, p < 0.001). The parameters of the gamma distribution can be used not only for identifying IOP induced changes in the optical scattering within the corneal stroma, but also in corneal geometry. The approach of corneal speckle analysis could be potentially utilized for an indirect and noninvasive assessment of some properties of corneal stroma.

## Introduction

Imaging corneal stroma is of interest in many ophthalmological applications, as stroma is essential for maintaining the shape and transparency of cornea [1] and, subsequently, for its refractive properties. Evaluation of corneal microstructure is usually associated with experimental microscopic and nanoscopic techniques such as scanning electron microscopy [2], X-ray scattering [3] and second-harmonic generation microscopy [4–7]. As noted by Tan et al. [8], it is important for any such imaging method to provide information on the corneal stroma without employing histological or labeling procedures.

Continuing advances in the technology of optical coherence tomography (OCT) have pushed the boundary between macroscopic and microscopic corneal imaging, bringing the axial resolution of the acquired data to about one micrometer [9–11]. Translating such advancements to the clinical practice appears to have a quick developmental path, despite that OCT currently lacks the specificity and high resolution of other experimental imaging methods, to image collagen organization of the corneal stroma *in-vivo*.

Recently, it has been demonstrated that some properties of the corneal microstructure can be indirectly assessed with OCT by exploring the statistical properties of speckle [12,13]. In particular, it has been anticipated that some distributional parameters of the corneal OCT speckle image intensities are related to the corneal scatterer cross section whereas others to the scatterer density. Those parameters could be indirectly linked to the collagen fibril organization in the stroma. In this particular OCT approach, the speckle, resulting from random interference of mutually coherent waves, is viewed as the source of information [14], where the techniques for suppressing it, otherwise viewed as constructive [15], are avoided. To date, it has been shown that by statistically modeling the corneal OCT speckle, it is possible to assess age-related changes to corneal stroma or short-term changes occurring during corneal swelling [12]. Further, a weak but statistically significant correlation between uncorrected intraocular pressure (IOP) tonometry-based measurements and statistical parameters of speckle was established for a group of healthy subjects with normal IOP values [16]. Also, analyzing corneal OCT speckle has shown some potential in glaucoma study, where the relationship between corneal scatterer cross section and scatterer density was similar in groups of glaucoma suspects and glaucoma patients but evidently different from that exhibited by healthy controls [17].

To apply the speckle-based approach of assessing corneal stroma properties to a clinical practice, a number of confounding factors that may influence the speckle statistics need to be removed from consideration. Of particular interest is the estimation of influence of *true* IOP and the ocular biometry on the speckle pattern. There are different algorithms aiming at relating indirect tonometry measurements of IOP with its true value occurring in the eyeball [18,19]. However, until now, the *true* IOP can only be measured invasively by inserting into the eye a needle connected to a pressure gauge. Consequently, many studies interested in the evaluation of *true* IOP, and particularly those aiming at assessing the effect of IOP on its tonometry based measurement [20], resort to the so-called inflation tests. In recent studies such inflation tests were combined with OCT imaging to facilitate the assessment of biomechanical properties of the cornea [21,22]. Nevertheless, in those works only the information on corneal geometry, such as the position of corneal apex, its radius of curvature and thickness, was taken into account from OCT, but not that on the structure of corneal stroma.

Further, the whole eye inflation testing, conducted *ex-vivo*, has some limitations referring, for example, to the duration of an experiment, inherent changes in hydration of ocular tissues or their biomechanical responses [23]. Hence, relevant settings of the *ex-vivo* experimental conditions are crucial to minimize any bias that could affect the results of assessing the properties of corneal stroma.

Development of methods of non-destructive and *in-vivo* quantifying corneal microstructure is of clinical significance. It is of interest whether the indirect speckle-based approach of OCT can be used to identify changes in the corneal stroma associated with IOP increase or alterations in corneal geometry with some precision. Hence, the aim of this work is to assess the effect the precisely controlled IOP may have on the corneal OCT speckle distribution and to what extent such relationships are affected by corresponding changes in ocular biometry. For this, an *ex-vivo* inflation experiment of intact porcine eyes, is considered, regarding also the influence of experiment’s duration on evaluating corneal speckle pattern.

## Materials and methods

### Experimental setup

Porcine eyes from domestic pigs (*sus scrofa domestica*) were obtained immediately after slaughter from a local abattoir (Meat Processing Plant, Otmuchow, Poland, accredited), stored in a medium of phosphate-buffered saline (PBS) solution, and transported to the laboratory in a portable refrigerator at 4°C. Then, the residues of the eye muscles were removed and the eyes were examined for inclusion in the study.

The inclusion criteria to qualify an eye to the measurements were: lack of mechanical damage of the eyeball, lack of corneal edema or endothelial damage, as well as corneal transparency that was carefully examined using a slit-lamp biomicroscope. The eyeball was placed in a custom-designed holder to limit movements and rotations of the eye during measurements. Additionally, the anterior–posterior eye movements were minimized by gently connecting the optic nerve to the post (located outside the holder) with a polyamide sewing thread. The inner part of the holder was padded with the cotton moistened with PBS. The same solution was used to hydrate the eyeball surface regularly during the measurements.

**Fig 1:**
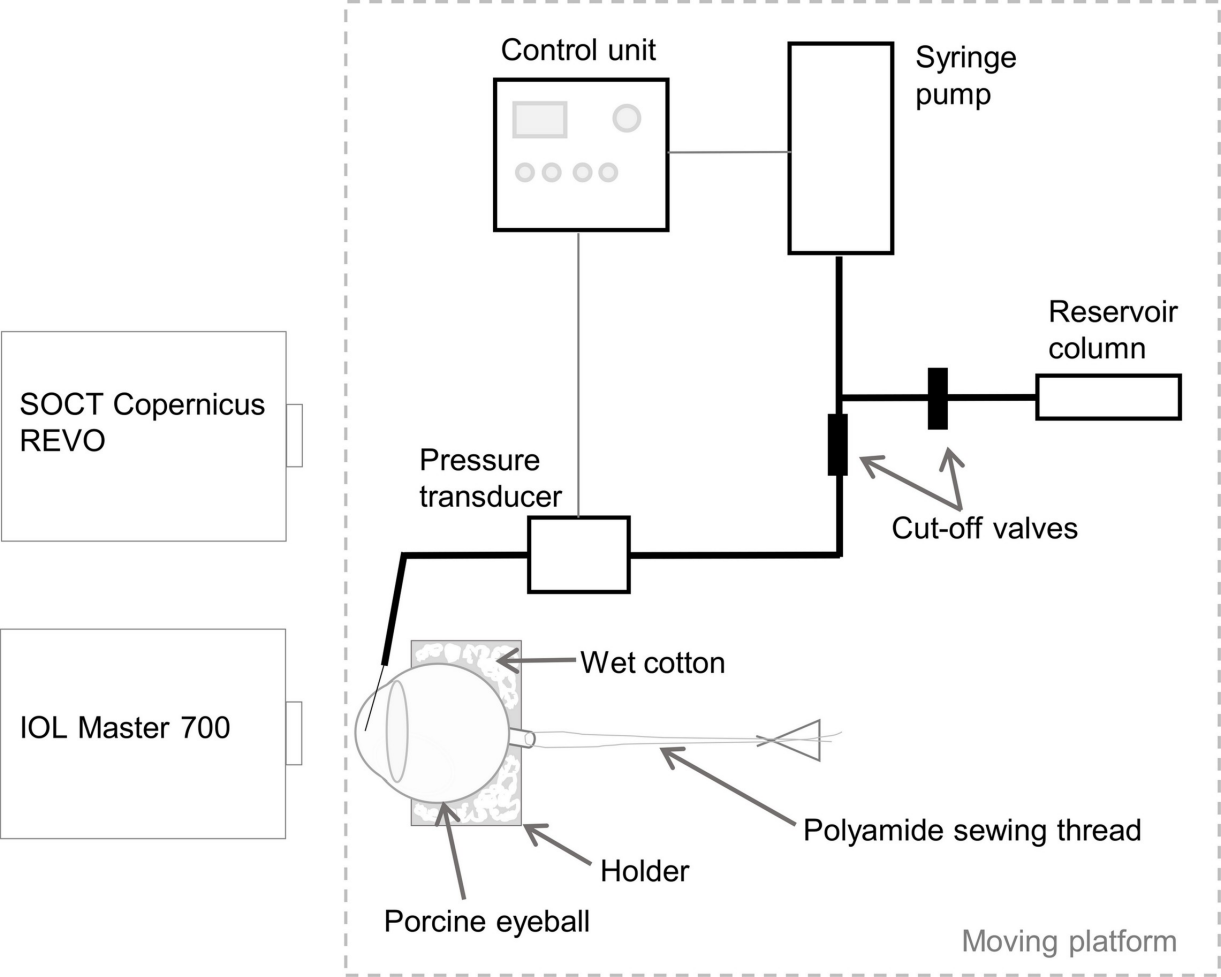
A scheme of the setup for the measurements of the corneal OCT speckle and the ocular biometry in the inflation test (Experiment 1) and at the constant IOP level (Experiment 2).

Two experiments were considered in this study: the inflation test (Experiment 1) and the examination at constant IOP level (Experiment 2). Both experiments were conducted in a setup similar to that described previously [24] in terms of fluid delivery, but heavily modified with regard to control of IOP. In this study, IOP in the anterior chamber of the eye was continuously set and adjusted by a custom-made control system (see Fig 1), whose purpose was to maintain high precision and accuracy of the *true* IOP. The experimental setup consists of a 20-gauge needle inserted into the anterior chamber through the corneo-scleral area and connected by tubing to a WIKA P-30 precision pressure sensor (WIKA Alexander Wiegand SE & Co. KG, Klingenberg, Germany), as well as a microinfusion closed-loop syringe pump connected by tubing to the pressure sensor and by tubing via a three-way stopcock to a reservoir column filled with PBS. The custom-made IOP control system consists of a glass piston pump, a screw gear with stepper motor, a pressure sensor, a control module with operator panel, a stepper motor controller and a power supply. The fluid is pumped to the eye by linear movement of the pump piston, driven by a stepper motor through the gear. The motor is driven using digital signals from the control module with a dedicated central unit, realized on a Cortex-M4 180 MHz ARM microprocessor, enabling micro-steps and precise positioning. The control software was written using the C language. The change or maintenance of pressure in the eyeball is carried out in a feedback loop. The control software retrieves current information from the pressure sensor and determines the motor and pump movement based on the requested pressure value and PID (proportional–integral–derivative controller) parameters. The system allowed for a precise change in pressure from several to several dozen mmHg. The whole IOP control system and the eyeball holder were mounted on a custom-designed XY-linear translation platform that enabled positioning the eye to one of the measuring devices.

In Experiment 1, for each of the eyeball tested, the IOP level was increased from 10 mmHg to 40 mmHg in steps of 5 mmHg. At each set IOP value, the infusion/withdrawal PBS volume was automatically adjusted by the control system for the *true* IOP to reach a desired value. A 3-minute break was undertaken before proceeding to ensure stable levels of pressure. Following this, ocular biometry was measured using the IOL Master 700 (Carl Zeiss Meditec AG, Jena, Germany). The following parameters were considered: central corneal thickness (CCT), axial length (AL), anterior chamber depth less CCT (AQD), and the keratometry parameters, translated into power vectors (M–mean equivalent spherical power, and J0 and J45 –astigmatic components of Jackson cross-cylinder), using the method of Salmon and Thibos [25]. After biometry, at each level of set IOP, three single (non-averaged) B-scans of central 5 mm cornea were acquired using spectral OCT (SOCT Copernicus REVO, Optopol, Zawiercie, Poland). The center wavelength, half bandwidth, axial and transversal resolutions of the device are 830 nm, 50 nm, 5 μm and 15 μm, respectively. The scanning speed is 80 000 A-scans per second. Each B-scan of size 1536×736 pixels was registered with the highest available resolution (12032 A-scans). To minimize the effect of OCT beam focus on speckle statistics, all measurements were acquired at a constant aperture within the bands of the instrument’s depth of focus using the instrument’s own guiding system.

To examine the influence of experimental conditions, particularly duration of the experiment, on the OCT speckle statistics, the second experiment was performed (Experiment 2). Another set of porcine eyes that met the inclusion criteria were examined in the described above setup, but this time at the constant IOP level of 15 mmHg, which was found as the typical value of pressure inside the pig’s eye [26]. Registration of ocular biometry and OCT corneal speckle was conducted in the same manner as in Experiment 1. The entire measurement cycle for all seven considered levels of IOP in Experiment 1 took about 70 minutes. The duration of Experiment 2 was set equally, which resulted in taking measurements at constant IOP every 10 minutes (t_1_, t_2_, …, t_7_). Both experiments were conducted at average temperature 22,5°C (standard deviation ± 1,9°C), and average humidity of 50,2% (± 4,5%). All measurements were completed for each eyeball at the maximum of seven hours post-mortem.

Forty one eyeballs were included in the study. From those, 8 eyeballs had CCT outside the assumed population limit set from 800 μm to 950 μm (see [26]) and were excluded. Remaining 33 eyeballs were used for further examinations: 23 eyeballs for the IOP inflation test (Experiment 1) and 10 eyeballs for measurements at the constant IOP value of 15 mmHg (Experiment 2).

### Corneal speckle distribution

OCT images contain characteristic noise called speckle, which is in the form of grainy structures. Speckle in OCT may be treated as noise but also as an information carrier [14]. A variety of probabilistic models have been proposed for speckle modeling. Mcheik et al. [27] were studying the performance of Rayleigh, lognormal, Nakagami and generalized gamma distributions for speckle modeling in OCT images of skin layers and concluded that the generalized gamma distribution was the best for that purpose. Lindenmaier et al. [28] used the gamma distribution for differentiating normal skin tissue from that with tumor, whereas Grzywacz et al. [29] showed that the stretched exponential distribution is most suitable for characterizing the speckle in images of retinal layers. Further, Jesus and Iskander [12] concluded that generalized gamma distribution is the most suitable probabilistic model (among a set of considered models) for characterizing corneal stroma in images acquired with SOCT Copernicus (a predecessor of OCT REVO used here). Additionally, the generalized gamma model was used there for log-transformed images, a typical procedure used to better visualize the tissue structures, whereas the use of B-scan in a raw format is preferred. In the current study, the gamma distribution was chosen to model the corneal OCT speckle in images which were previously preprocessed using inverse log transformation. The proposed model is more robust than its generalization, and has a strong theoretical basis [30]. Gamma distribution is a two-parameter distribution with probability density function:

$$f_{G}(x;a,v)=\frac{1}{\Gamma (v)a^{v}}x^{v-1}e^{-\frac{x}{a}}\dot{=}\Gamma (a,v),$$

where *a* is the scale parameter and *v* is the shape parameter. Gamma distribution facilitates interpretation of the parameters, as compared to the generalized gamma distribution, in which two shape parameters are nonlinearly dependent [31]. Tunis et al. [32] used generalized gamma distribution for modeling the speckle distribution in ultrasound images and suggested that the two shape parameters may be related to the effective scatterer number density, whereas the scale parameter can reflect the average scatterer cross-section. A similar conclusion has been reached by Lindenmaier et al. [28] when applying gamma distribution to the OCT speckle of cancer images.

### Data processing

**Fig 2:**
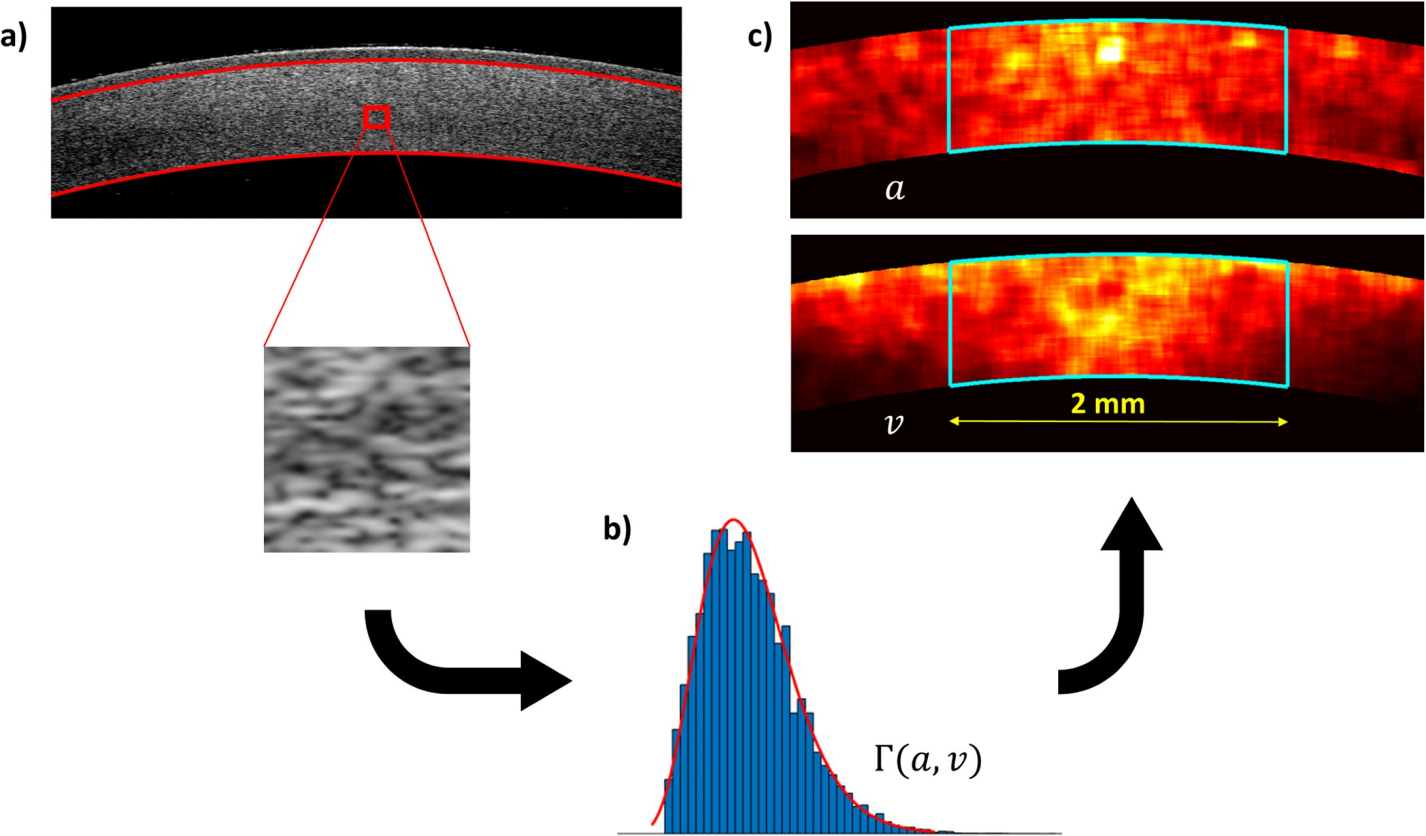
The process of generating spatial maps of the parameters of gamma distribution within the corneal stroma. The scanning window moves through the OCT scan of the cornea, where the profile of the posterior cornea as well as that of the Bowman’s layer (red solid lines) are delineated first (a). For each window position, the parameters of gamma distribution are estimated from the pixel intensities within the window (b). Next, estimates of the parameters are set in the spatial maps as values of the central pixel of the window, hence creating the spatial maps of the parameters (c). The region of interest is indicated by cyan lines on the maps and it includes the entire thickness (vertical span) of the stroma.

All calculations and analyses were performed in MATLAB (MathWorks, Inc. Natick, MA, USA). Firstly, the log transformation, applied automatically to every OCT B-scan in the device software, was inversed. Secondly, delineation of anterior and posterior corneal profiles, as well as that of Bowman’s layer, was achieved with the method described earlier [12]. Spatial maps of the parameters of gamma distribution were created for the corneal stroma using a scanning window of size 41×41 pixels by sliding it with a step of 10 pixels within the area encapsulated by the posterior corneal profile and that of the Bowman’s layer. The size of the scanning window was chosen empirically as a trade-off between sufficiently high resolution of the maps and sufficiently large sample size, to reliably estimate the distributional parameters. The parameters of the gamma distribution model were estimated, using the method of maximum likelihood [33], for pixel intensities within the scanning window and their estimates were set in the distribution maps as a value of the central pixel of the window. Fig 2, shows schematically the process of generating the spatial maps of the parameters of gamma distribution for the corneal stroma. The region of interest (ROI) was then selected from the spatial maps of parameters (Fig 2C). The ROI was set within the central 2 mm horizontal span of the stroma [12], to avoid the potential bias of corneal curvature changes. Within the ROI, the mean values for each of the two parameters of gamma distribution (*a, v*) were calculated and used for further analysis. Note that the sliding step of 10 pixels mentioned above was an empirically reached compromise between the resolution and the effect of correlation length on the standard inaccuracy of the means of the gamma distribution parameters.

All experimental data are included in the supplementary file S1 File of Supporting information.

### Statistical analysis

Gamma distribution parameters as well as parameters from ocular biometry were taken into consideration in the statistical analysis. One-way repeated measures analysis of variance (ANOVA) was applied to investigate whether all considered parameters vary with IOP (Experiment 1) or with time (Experiment 2). Next, the post-hoc analysis was used to assess the differences in mean values of all parameters between different levels of IOP or between consecutive time points. Correction was not used in the post-hoc analysis because a large number of tests (i.e., 21 for 7 considered levels of IOP) were carried out without a preplanned hypothesis [34]. Further, partial correlation analysis with IOP set as a control variable in Experiment 1 and with time (*t*) set as a control variable in Experiment 2, was performed for the parameters of gamma distribution and ocular biometry.

When testing for zero correlation, the sample size of 23 used in this study in Experiment 1 and of 10 in Experiment 2 deemed significant correlation values (in absolute terms) those that are larger than 0.6 and 0.8, respectively [35]. Nevertheless, in this study absolute correlation values close to 0.6 in Experiment 1 and close to 0.8 in Experiment 2 for which the test power falls just below 80% are also considered significant.

## Results

**Fig 3:**
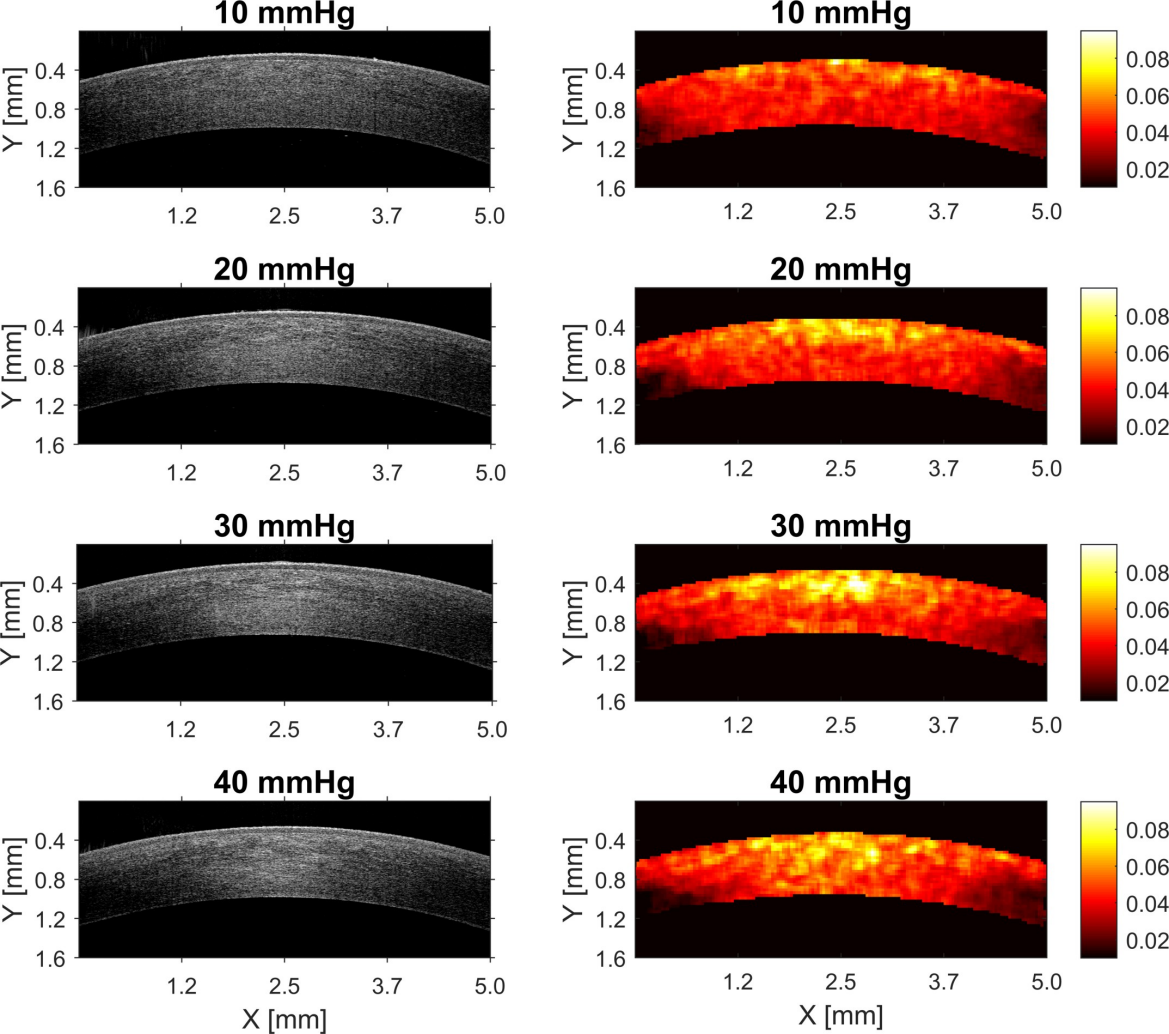
Illustrative spatial maps of the scale parameter (*a*) of gamma distribution and the corresponding OCT scans for four out of seven values of IOP in Experiment 1.

**Fig 4:**
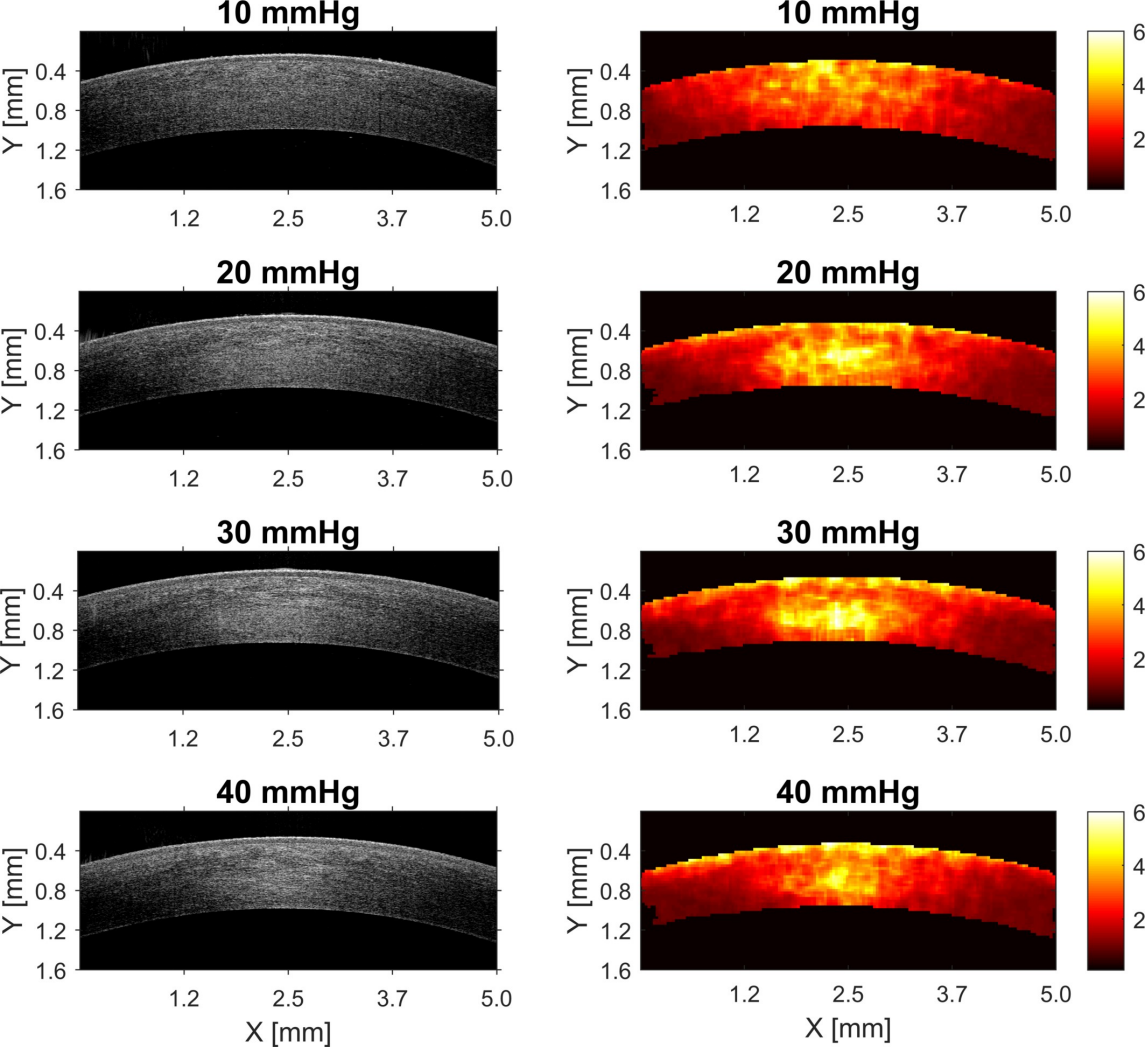
Illustrative spatial maps of the shape parameter (*v*) of gamma distribution and the corresponding OCT scans for four out of seven values of IOP in Experiment 1.

The analysis was performed based on spatial maps of the parameters of gamma distribution. Illustrative spatial maps are presented in Figs 3 and 4, for a set of IOP values ranging from 10 mmHg to 40 mmHg changing in steps of 10 mmHg. Some changes in the distribution of the spatial maps of gamma parameters related to IOP elevation are visually recognizable.

**Fig 5:**
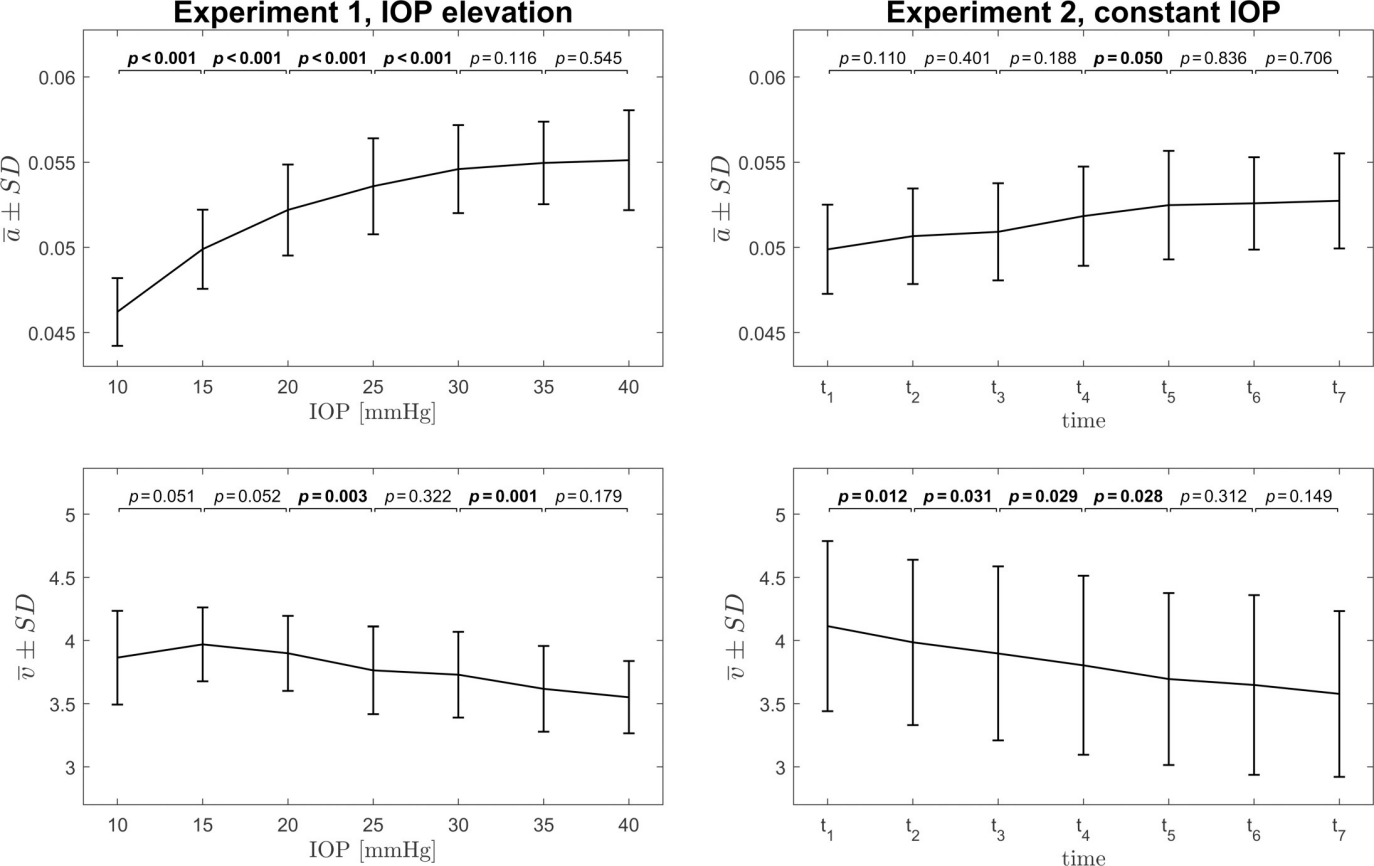
Plots of the group mean values of the parameters of gamma distribution as a function of set IOP for the Experiment 1 (left column) and as a function of time in Experiment 2 (right column). The statistical significance of differences between the parameters of gamma distribution for the adjacent IOP levels or consecutive time points, assessed using paired t-test, is presented above the plots. Statistically significant results are bolded.

Mean values of the parameters of gamma distribution were calculated for the considered ROI (see Fig 2c) for each of the three measurements of each eye at the given condition and the median value from those measurements was taken as the parameter estimate to be tested within the group. One-way repeated measures ANOVA performed for this data showed that both parameters change statistically significantly in Experiment 1 and 2 (all *p*-values < 0.001). Fig 5 shows the group mean values of those parameters together with the standard deviation bars for all considered set values of *true* IOP, or time points, separately for the Experiment 1 and 2. There is a clear dependence of the scale parameter (*a*) on IOP values with faster increase at the lower levels of IOP (up to 25 mmHg) and a saturation phase for the higher IOP values. Differences in the *a* values are statistically significant, mostly for the lower values of IOP (indicated by *p*-values in Fig 5). Such relationship is not observed for the scale parameter in Experiment 2, where IOP is kept constant at the level of 15 mmHg. Also, almost all differences in values of parameter *a* between the adjacent IOP levels are not statistically significant. Moreover, it is worth noting that the mean values of the scale parameter (*a*) in the corresponding points in both experiments (IOP = 15 mmHg in Experiment 1 and t_2_ in Experiment 2) are coincident. The shape parameter (*v*) generally decreases with IOP and time. Nevertheless, a rise of the *v* value is observed in Experiment 1 between 10 mmHg and 15 mmHg.

**Fig 6:**
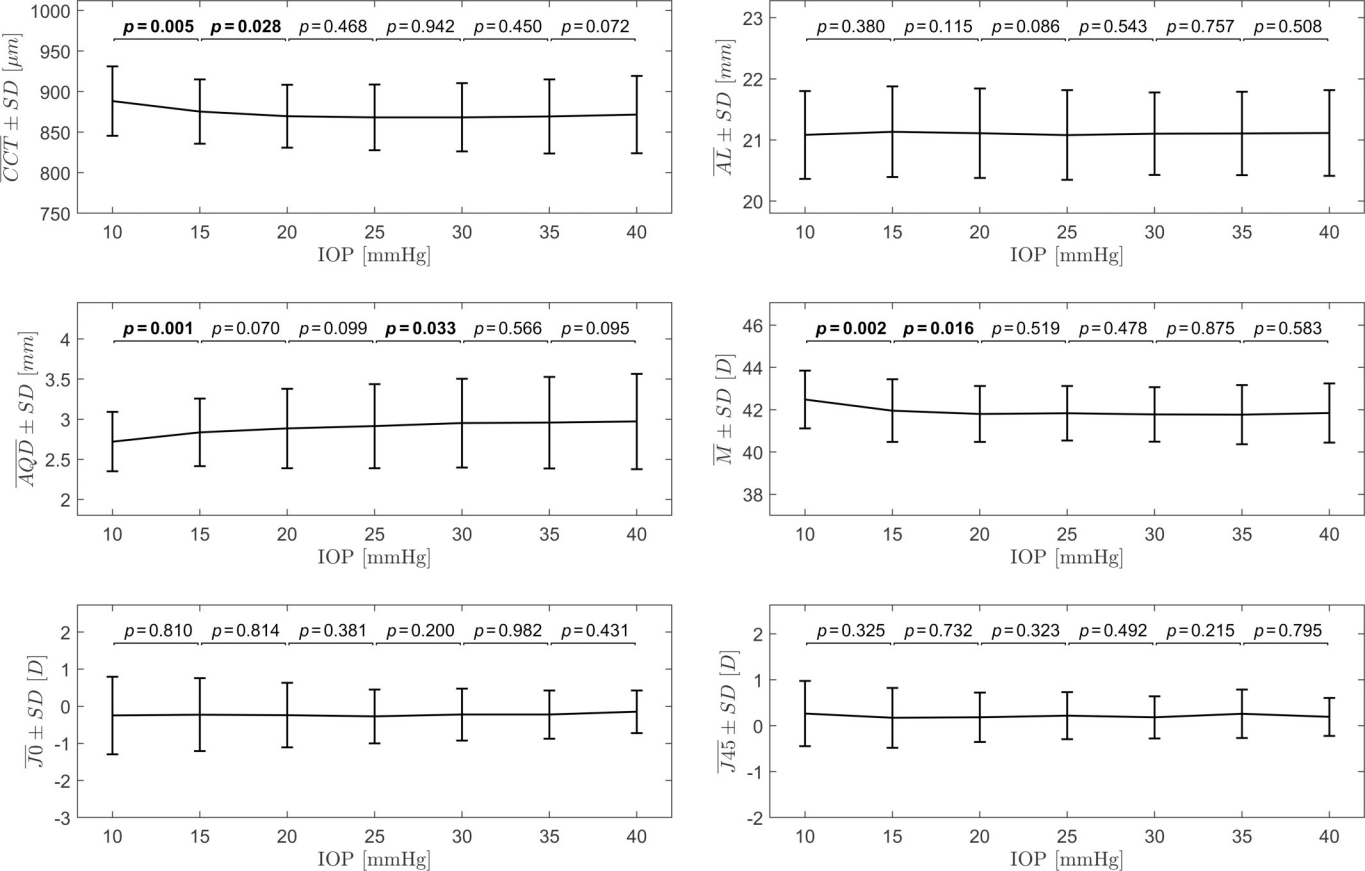
Plots of the group mean values (with standard deviations) of the ocular biometric parameters as functions of set IOP for the Experiment 1. The statistical significance of differences between the parameters for the adjacent IOP levels, assessed using paired *t*-test, is presented above the plots. Statistically significant results are bolded.

**Fig 7:**
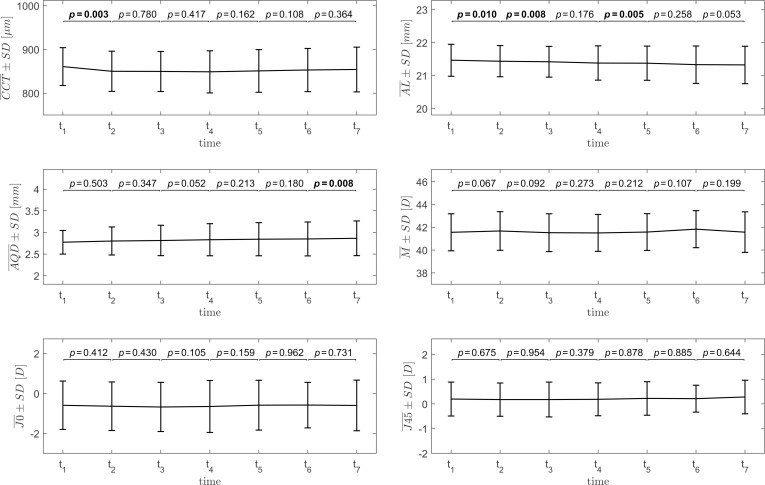
Plots of the group mean values (with standard deviations) of the ocular biometric parameters as functions of time for the Experiment 2. The statistical significance of differences between the parameters for consecutive time points, assessed using paired *t*-test, is presented above the plots. Statistically significant results are bolded.

Group mean values of the ocular biometry parameters assigned at different levels of *true* IOP or different time points are gathered in Tables 1 and 2, respectively. Results of one-way repeated measures ANOVA indicate that CCT, AQD and M change statistically significantly with IOP (Experiment 1), whereas in Experiment 2 only CCT and AL change statistically significantly with time. The results of post-hoc analysis for ocular biometry parameters for Experiment 1 and 2 are presented on the plots in Figs 6 and 7, respectively.

**Table 1:** Experiment 1.

Set IOP [mmHg]		CCT [μm]	AL [mm]	AQD [mm]	M [D]	J0 [D]	J45 [D]
10		888 ± 43	21.1 ± 0.7	2.72 ± 0.37	42.5 ± 1.4	−0.2 ± 1.0	0.3 ± 0.7
15		875 ± 40	21.1 ± 0.7	2.84 ± 0.42	42.0 ± 1.5	−0.2 ± 1.0	0.2 ± 0.7
20		870 ± 39	21.1 ± 0.7	2.88 ± 0.50	41.8 ± 1.3	−0.2 ± 0.9	0.2 ± 0.5
25		868 ± 40	21.1 ± 0.7	2.91 ± 0.52	41.8 ± 1.3	−0.3 ± 0.7	0.2 ± 0.5
30		868 ± 42	21.1 ± 0.7	2.95 ± 0.55	41.8 ± 1.3	−0.2 ± 0.7	0.2 ± 0.5
35		869 ± 46	21.1 ± 0.7	2.96 ± 0.57	41.8 ± 1.4	−0.2 ± 0.6	0.3 ± 0.5
40		872 ± 48	21.1 ± 0.7	2.97 ± 0.59	41.8 ± 1.4	−0.1 ± 0.6	0.2 ± 0.4
**ANOVA results**	***F***	**3.24**	0.45	**7.44**	**9.33**	0.32	0.38
	***p***	**0.005**	0.841	**< 0.001**	**< 0.001**	0.928	0.888

IOP—intraocular pressure; CCT—central corneal thickness; AL—axial length; AQD—anterior chamber depth less CCT; M—mean equivalent spherical power; J0 and J45—astigmatic components of the Jackson cross-cylinder.

Group mean (± one standard deviation) values of the ocular biometry parameters at different IOP levels and the results of one-way repeated measures ANOVA. Statistically significant results are bolded.

**Table 2:** Experiment 2.

Time		CCT [μm]	AL [mm]	AQD [mm]	M [D]	J0 [D]	J45 [D]
t_1_		861 ± 43	21.5 ± 0.5	2.77 ± 0.27	41.6 ± 1.6	−0.6 ± 1.2	0.2 ± 0.7
t_2_		850 ± 46	21.4 ± 0.5	2.80 ± 0.32	41.7 ± 1.7	−0.6 ± 1.2	0.2 ± 0.7
t_3_		850 ± 46	21.4 ± 0.5	2.81 ± 0.35	41.5 ± 1.7	−0.7 ± 1.2	0.2 ± 0.7
t_4_		849 ± 48	21.4 ± 0.5	2.83 ± 0.37	41.5 ± 1.5	−0.7 ± 1.2	0.2 ± 0.6
t_5_		851 ± 49	21.4 ± 0.5	2.84 ± 0.38	41.6 ± 1.6	−0.6 ± 1.2	0.2 ± 0.7
t_6_		853 ± 49	21.3 ± 0.6	2.85 ± 0.39	41.8 ± 1.6	−0.6 ± 1.1	0.2 ± 0.5
t_7_		854 ± 51	21.3 ± 0.6	2.86 ± 0.40	41.6 ± 1.7	−0.6 ± 1.2	0.3 ± 0.6
**ANOVA results**	***F***	**2.39**	**5.15**	1.48	1.44	0.52	0.69
	***p***	**0.04**	**< 0.001**	0.203	0.218	0.792	0.659

CCT—central corneal thickness; AL—axial length; AQD—anterior chamber depth less CCT; M—mean equivalent spherical power; J0 and J45—astigmatic components of the Jackson cross-cylinder.

Group mean (± one standard deviation) values of the ocular biometry parameters at different time points and the results of one-way repeated measures ANOVA. Statistically significant results are bolded.

Tables 3 and 4 present the values of partial correlation coefficient *r* and the corresponding *p*-values between the parameters of gamma distribution and the parameters of ocular biometry.

**Table 3:** Partial correlation coefficients for the scale (*a*) and shape (*v*) parameters of gamma distribution and ocular biometry parameters with IOP set as control variable for Experiment 1.

	**CCT**	**AL**	**AQD**	**M**	**J0**	**J45**
***a***	***r* = −0.576**	*r* = −0.073	*r* = 0.003	*r* = −0.100	*r* = −0.124	*r* = −0.269
	***p* < 0.001**	*p* = 0.361	*p* = 0.973	*p* = 0.209	*p* = 0.119	*p* = 0.001
***v***	*r* = −0.009	*r* = 0.215	*r* = −0.164	*r* = −0.129	*r* = 0.169	*r* = −0.170
	*p* = 0.906	*p* = 0.006	*p* = 0.038	*p* = 0.105	*p* = 0.033	*p* = 0.032

CCT—central corneal thickness; AL—axial length; AQD—anterior chamber depth less CCT; M—mean equivalent spherical power; J0 and J45—astigmatic components of the Jackson cross-cylinder.

Bolded results indicate that for the sample size of 23 used in this Experiment the absolute correlation values are close to 0.6 for which the test power is close to 80% for considering them significant.

**Table 4:** Partial correlation coefficients for the scale (*a*) and shape (*v*) parameters of gamma distribution and ocular biometry parameters with time set as control variable for Experiment 2.

	CCT	AL	AQD	M	J0	J45
***a***	*r* = −0.425	*r* = −0.208	*r* = −0.228	*r* = 0.100	*r* = 0.008	*r* = 0.453
	*p* < 0.001	*p* = 0.086	*p* = 0.060	*p* = 0.420	*p* = 0.948	*p* < 0.001
***v***	*r* = 0.420	*r* = 0.312	*r* = 0.136	*r* = −0.207	*r* = 0.223	*r* = −0.358
	*p* < 0.001	*p* = 0.009	*p* = 0.265	*p* = 0.092	*p* = 0.058	*p* = 0.003

CCT—central corneal thickness; AL—axial length; AQD—anterior chamber depth less CCT; M—mean equivalent spherical power; J0 and J45—astigmatic components of the Jackson cross-cylinder.

Bolded results indicate that for the sample size of 10 used in this Experiment the absolute correlation values are close to 0.8 for which the test power is close to 80% for considering them significant.

Taking into account sample size requirement for one correlation test with power of 80% and α = 0.05 [35], only one statistically significant correlation between the scale (*a*) parameter and CCT in Experiment 1 can be considered (see Table 3).

## Discussion

Elevation of IOP is not the only factor that can influence stromal microarchitecture and hence possible changes in the corneal OCT speckle statistics. Experimental environment, including but not limited to primarily duration of experiment, temperature, humidity, type of solution used for moistening and eyeball filling, as well as post mortem process, all have the impact on the state of the examined corneal tissue. To differentiate the effect of experimental conditions from the influence of IOP elevation on the corneal OCT speckle statistics, two experiments were proposed in this study (Experiment 1 and 2). Conditions of their conduction were analogous, but the IOP values were changing only in Experiment 1. It resulted in the possibility of estimation of the effect of IOP elevation by itself on the speckle distribution.

Spatial distribution of the parameters of the gamma model of corneal OCT speckle, in forms of the maps shown in Figs 3 and 4, can be used to indirectly assess the changes in the corneal stroma due to increase in IOP. As the first approach, basic description in a form of the mean parameter of the spatial map was used for the analysis, when intact porcine eyes were subjected to the increase of precisely controlled *true* IOP in the anterior chamber.

A change in IOP alters properties of the cornea such as its geometry, microstructure and biomechanical behavior [22,36,37] by acting on the orientation and the density of collagen fibrils, as well as their interspacing. In particular, it was shown using nonlinear optical microscopy that interlamellar gaps decreased in size with increasing IOP in isolated rabbit corneas [38]. Also, in the rats’ study, the reduction of the corneal collagen fibril diameter was observed after IOP elevation [39]. Furthermore, investigation of the human stroma, subjected to the elevated IOP, revealed reorganization of lamellae in the stroma [7]. For postmortem porcine eyes, strong influence of IOP on the second-harmonic reflection imaging of the corneal stroma was observed [37]. This study supports those findings as changes in the corneal OCT speckle statistics were observed during the inflation experiment on enucleated intact porcine eyeballs. The results indicate that the increase in the IOP has an impact on the backscattered light, which, in turn, can indirectly reflect the variations in the arrangement of corneal stroma. Specifically, the average value of the *a* parameter of gamma distribution, used as a model for the corneal OCT speckle, has changed statistically significantly with the increasing IOP, whereas this value has remained stable throughout the experiment with constant IOP.

**Fig 8:**
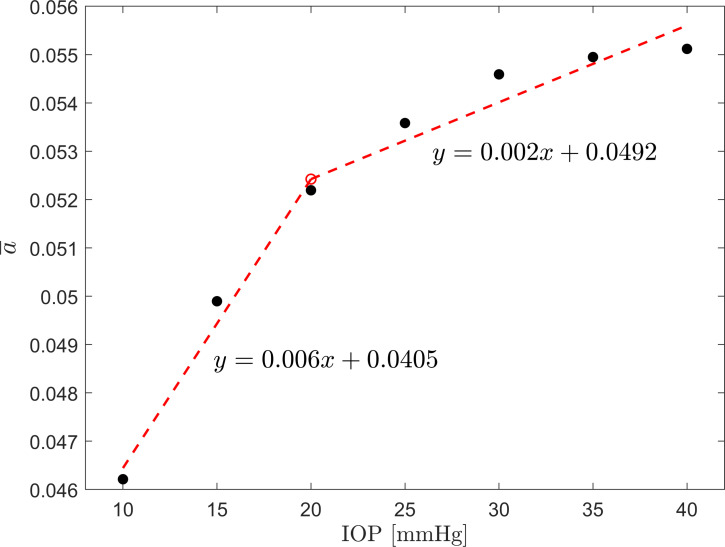
Plot of the group mean of the scale parameter (*a*) of the gamma distribution. Red dashed lines show the result of a constrained bi-linear model fitted to the data, with corresponding equations for each of the lines. The transition point is estimated at 20 mmHg.

Structural analysis of the corneal behavior, under different loading states, led to the two-phase model of stroma reaction to increasing IOP, i.e., a matrix-regulated phase and a collagen-regulated phase [40]. Anderson et al. explained that at lower IOPs cornea’s behavior is dominated by the corneal matrix (stroma), whereas collagen fibril layers remain loose and are unable to notably contribute to the overall performance. In the second phase, it is expected that the fibril layers become taut and due to their much higher stiffness they start to control the overall stroma behavior. In this study, similar two trends are noticed when analyzing the behavior of the *a* parameter when IOP is elevated. A constrained bi-linear model estimated using an iterative least-squares algorithm [41], where the estimate of the second line is conditioned on the estimate of the first line, fitted to the average values of the scale parameter (*a*), revealed a transition point at 20 mmHg (see Fig 8).

Changes in values of ocular biometry parameters during the inflation test is another interesting observation. Of particular interest are the changes in CCT, which may be also an indicator of undesirable corneal swelling. Hatami-Marbini et al. [42] showed that swelling behavior is similar for human and porcine corneas and that the corneal thickness is strongly dependent on its hydration. They have also pointed out that different hydration solutions may influence the biomechanical behavior of the corneal sample that is examined [43]. Therefore, in this study, to prevent excessive cornea swelling, regular hydrating of the eyeballs was provided during the experiment. The results indicate that there was a slight decrease in CCT at 25 mmHg, on average about 2.2% in Experiment 1 and about 1.4% in Experiment 2. After that, CCT increased, but finally it did not reach the initial value at the beginning of the experiments (see Tables 1 and 2). Those small changes in CCT during the experiments suggest that use of PBS as a hydrating solution, although found not to be optimal in other studies [44], was here justified.

In addition, in Experiment 1, where IOP was elevated, a statistically significant decrease in CCT was observed for IOP values from 10 mmHg to 20 mmHg. Similar observation was described in the study by Wu et al. [38], where for isolated rabbit corneas CCT was also observed to decline on pressurization to 20 mmHg. Furthermore, in the current study, in Experiment 2 with the constant IOP, the decrease in CCT was less pronounced than that obtained in Experiment 1 and comparable with the study of [45], where small changes in CCT were observed for the untreated porcine eyes from the control group with IOP maintained at the level of 20 mmHg for 120 minutes. In the present study, it is anticipated that the effect of CCT decrease is not only a consequence of the IOP elevation (as it was shown in Experiment 1), but there is also an adjustment of the eyeball to the experimental conditions, in particular in its initial time (between t_1_ and t_2_ in Experiment 2). Moreover, in Experiment 1, CCT affects values of the *a* parameter of gamma distribution since both parameters are statistically significantly correlated. Hence, it could be assumed that changes in the values of the scale parameter are induced by changes in CCT, rather than by IOP elevation. Nevertheless, the post-hoc analysis revealed that statistically significant increase in CCT occurs for IOP values less than 20 mmHg, whereas for the *a* parameter statistically significant changes are observed for IOP values less than 30 mmHg. It is therefore concluded that IOP elevation affects not only CCT, but also induces changes in the *a* parameter of the gamma distribution.

In Experiment 1 statistically significant changes in ocular biometric parameters were also observed for anterior chamber depth less CCT (AQD) and mean equivalent spherical power (M), a parameter associated with the curvature of the cornea. The increase in AQD with IOP is assumed to be the effect of direct injection of PBS into the anterior chamber of the eye. On the other hand, the M parameter did not change substantially, except for the first measurement at IOP of 10 mmHg—a value below the physiological normal level of IOP for porcine eye [46]. The results of this study are in agreement with those reported by Pierscionek et al. [47], where the statistically significant changes in corneal curvature were not observed for IOP values from 15 to 45 mmHg. Further, in Experiment 2, where constant IOP value of 15 mmHg was maintained, statistically significant changes in AQD and M were not observed.

In the elevation test (Experiment 1), no statistically significant changes in AL values were noticed, whereas it is known from other studies that changes in IOP values are supposed to be correlated with AL [48,49]. Also, some statistically significant changes in AL values were observed in Experiment 2, although the IOP value was constant in that case. It can be assumed that time dependent postmortem changes of AL, linked perhaps to the changing of hydration state of ocular tissues, could compensate for the effect of any changes in AL caused by IOP elevation.

This study has some limitations. Firstly, it concerns porcine corneas and despite many similarities between porcine and human eyes, no extrapolation of the OCT speckle results to other mammalian eyes can be judiciously made without further comparative studies. Secondly, the experimental setup involved placing the needle in the anterior chamber, hence comparison to other studies, where a needle is inserted to the eyeball via the optic nerve, is difficult. Also, the experiment involved intact eyeballs prohibiting comparison to other studies with tissue cut samples of the cornea, as boundary conditions may substantially affect the results of OCT speckle characteristics. The presented here whole eye inflation testing refers to the settled experimental conditions that do not accurately reflect natural physiological conditions of the eyeball *in-situ*, such as temperature, ocular tissue hydration, and the ocular pulsation. However, this is a commonly used *ex-vivo* mechanical testing method [23,50], in which special care was taken about ocular tissue hydration so that the tissue responses would closely correspond to that of their *in-vivo* counterpart. Further, the model of the speckle statistics was chosen here as the gamma distribution. Although meritoriously justified, it does not limit the consideration of another statistical model, which would be instrument specific.

Finally, one could envisage examining the behavior of corneal speckle parameters in an experiment where IOP is first increased and then decreased. However, as shown in Experiment 2 with constant IOP, the time factor in such experiments has to be taken into account, because the eyeball being examined undergoes continuing deterioration, despite being hydrated. Hence, in a lengthy procedure that would exceed two hours (e.g. to examine the full cycle of increasing and decreasing IOP), the factors related to the tissue deterioration would confound the results.

## Conclusions

Summarizing, this study proves the hypothesis that different IOP values produce different identifiable distribution of the corneal OCT speckle. The parameters of gamma distribution, used here as the model of corneal OCT speckle, can be potentially utilized to infer not only about the IOP induced alterations in the optical scattering pattern within the stroma but also about geometry of the cornea. These findings provide yet another exciting platform for the development of OCT technology.

## Supporting information

S1 File Data set. (XLSX)
